# Giant gastric lipoma mimicking well-differentiated liposarcoma

**Published:** 2012-09-23

**Authors:** Mohamed Moncef Hamdane, Ehsen Ben Brahim, Mériam belhaj Salah, Nooman Haouas, Ahmed Bouhafa, Achraf Chedly-Debbiche

**Affiliations:** 1 Department of pathology, Habib Thameur Hospital. 8 rue Aly Ben Ayed. 1008. Montfleury, Tunis, Tunisia; 2 Department of visceral surgery, Habib Thameur Hospital. 8 rue Aly Ben Ayed. 1008, Montfleury, Tunis, Tunisia

**Keywords:** Stomach, lipoma, liposarcoma, pathology, cytogenetics

## Abstract

Authors report the case of a 51-year-old man, presenting with epigastralgia of recent onset. Physical exam was unremarkable. Endoscopy revealed a large, ulcerated, submucosal, antral tumor. CT scan reveals an antral mass with fat attenuation. The patient underwent a total gastrectomy. Macroscopic examination identified in the antral wall a 9-cm, well-circumscribed, nodular lesion, with a yellow, greasy cut surface. On histological examination, the tumor was composed of a mature adipocytes proliferation, showing significant variation in cell size, associated to some lipoblasts. Nuclei were sometimes large, slightly irregular, but without hyperchromasia nor mitosis. Diagnosis of a well-differentiated liposarcoma was suspected and molecular cytogenetic analyses showed no MDM2 nor CDK4 gene amplification on fluorescent in situ hybridization. The diagnosis of lipoma was made. Twelve months following surgery, the patient is doing well.

## Introduction

Lipomas are relatively uncommon tumors in the gastointestinal tract, often located in the right colon [1]. Gastric lipomas are even more unusual with approximately 220 cases reported in the literature [2]. The diagnosis is strongly suggested by abdominal CT scan findings and is confirmed by histology. Histopathological diagnosis is usually easy. However, the tumor may sometimes undergo significant inflammatory changes leading to misdiagnosis of this lesion with a well-differentiated liposarcoma (WDLS).

## Patient and observation

**Figure 1:**
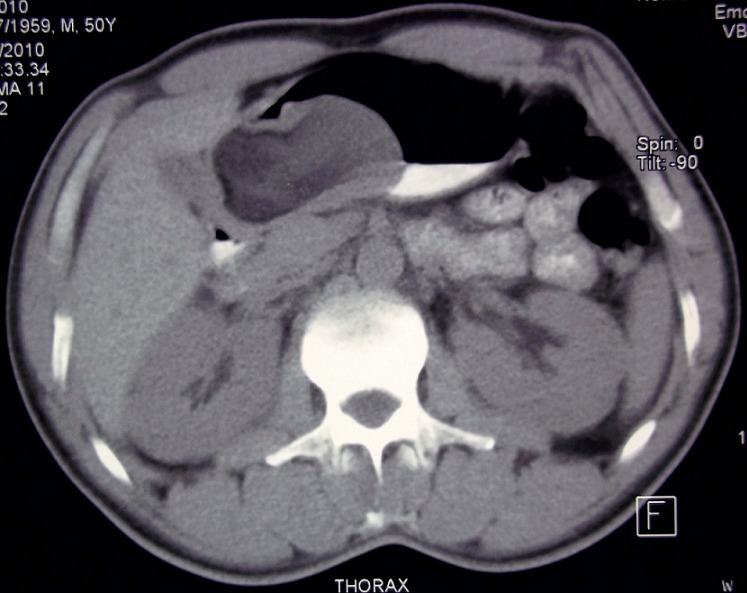
Abdominal CT scan: A large, low-attenuation, gastric mass

A 51-year-old man, in otherwise excellent general health, presented with epigastralgia of recent onset. The patient denied any history of gastrointestinal hemorrhage, nausea, vomiting, and change in bowel habits, fevers, or melena. Physical examination was unremarkable. Endoscopy revealed a soft, large, ulcerated, submucosal mass in the gastric antrum. Multiple biopsies were obtained but were all superficial, showing unspecific inflammation of the gastric mucosa. The abdominal CT scan revealed a round, well circumscribed, low-attenuation, gastric antral mass, measuring approximately 9 cm in diameter (Figure 1). The patient underwent total gastrectomy.

**Figure 2:**
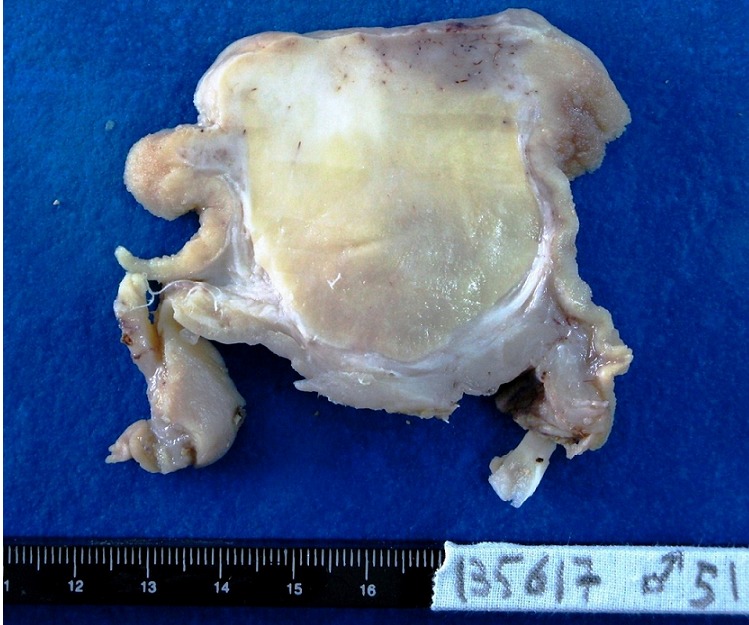
Macroscopic features: A well-circumscribed nodular lesion with a yellow, greasy, cut-surface

Gross examination of the surgical specimen identified in the antral submucosa, a well-circumscribed, smooth, nodular lesion, measuring 9x7.5x5 cm, with a homogeneous, yellow, greasy cut surface. Overlying mucosa was partially ulcerated (Figure 2).

**Figure 3:**
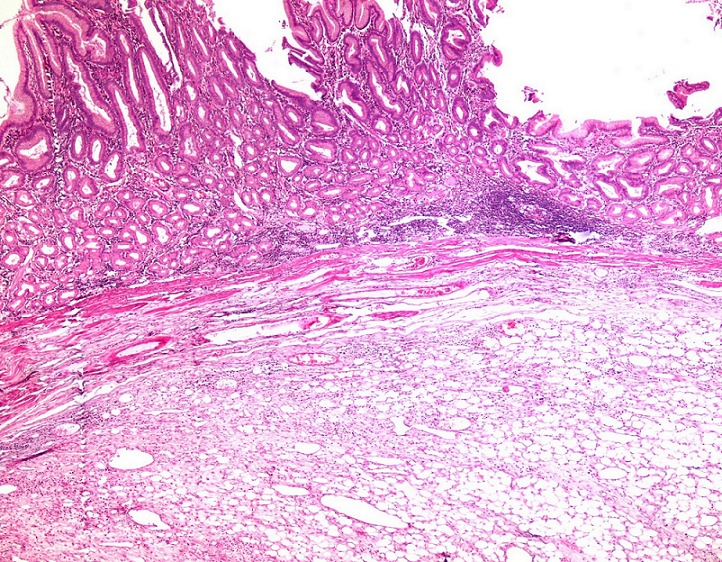
An adipocytic proliferation located in the submucosa (HEx40)

**Figure 4:**
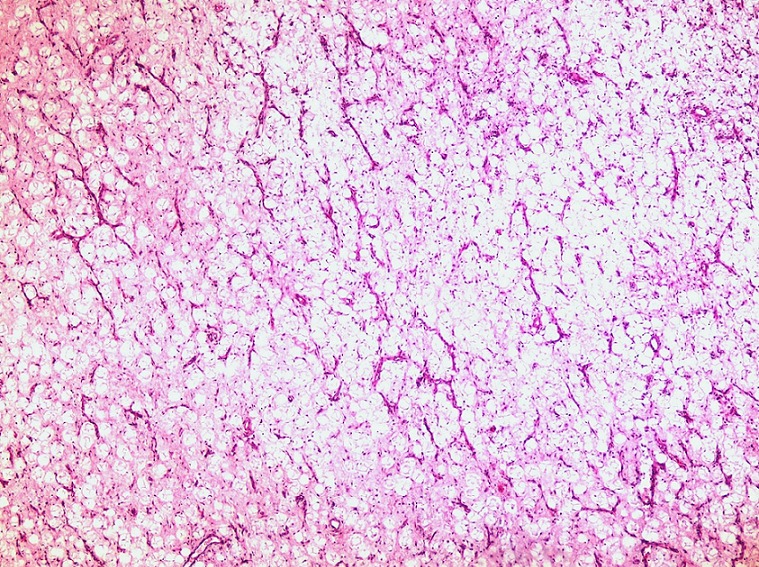
The tumor is made of an adipocytic proliferation showing a significant variation in cells size, with many branched capillaries, in a fibromyxoid background (HEx100)

**Figure 5:**
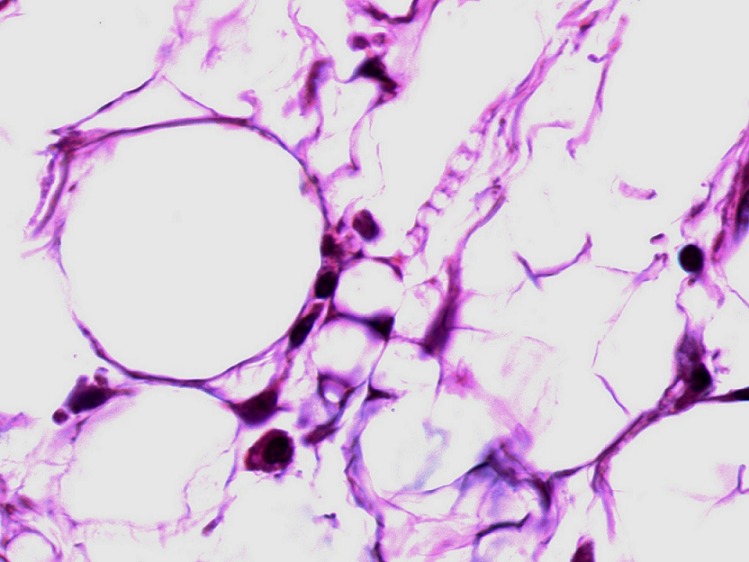
Lipoblasts are present within the adipocytic proliferation (HEx400)

**Figure 6:**
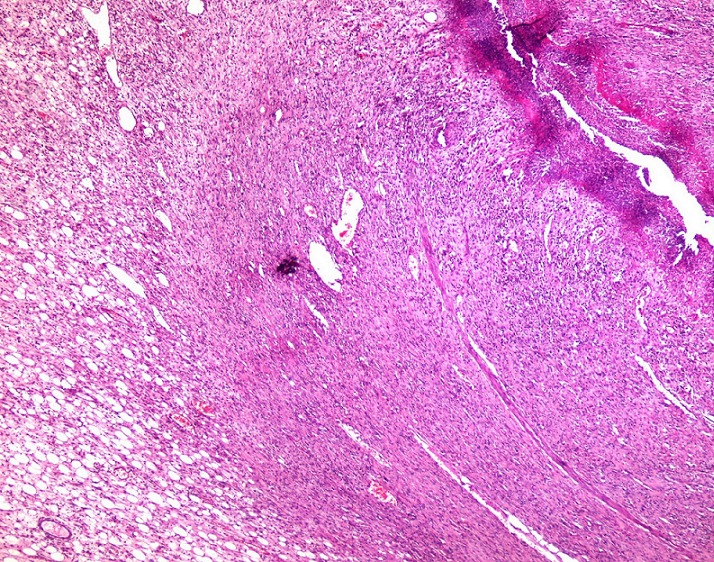
The overlying mucosa is focally ulcerated (HEx40)

Histological examination revealed a submucosal tumor (Figure 3), composed of a mature adipocytes proliferation, showing significant variation in cell size (Figure 4), associated to some lipoblasts (Figure 5), in a fibromyxoid background. Nuclei were sometimes large, slightly irregular, but without hyperchromasia nor mitosis. Many branching capillaries were seen. Areas of lower cellularity, displaying chronic inflammatory changes and containing bland spindle cells were also noted. The overlying mucosa was ulcerated (Figure 6) and the muscularis propria was focally dissociated by the tumor. The diagnosis of a WDLS was suspected. An immunohistochemical study was performed. Tumor cells were reactive with anti-HMGA2 and didn't express S-100 protein, CD34, MDM2 and CDK4. A cytogenetic study was then performed, showing no MDM2 nor CDK4 gene amplification on fluorescent in situ hybridization (FISH). The diagnosis of a benign lipoma was so made. The patient underwent an uneventful recovery. One year following surgery, he is doing well.

## Discussion

Gastric lipoma is a rare, benign lesion, accounting for only 5% of gastrointestinal tract lipomas and fewer than 1% of all gastric tumors [3, 4]. They typically occur in the fifth or sixth decade of life with equal sex incidence [2, 4, 5]. 75% of gastric lipomas are located in the antrum and are usually submucosal in origin [2].

Clinical symptoms depend on the size of the lesion. When small (< 2cm), lipomas are usually asymptomatic and they are often discovered fortuitously [1, 3, 4]. When the tumors are large (> 3 − 4cm), patients often present with upper gastrointestinal hemorrhage, either chronic or acute, caused by ulceration of the neoplasm [1, 4]. Abdominal pain and obstructive symptoms are also common, especially if there is endoluminal growth that could cause intussusception [1, 4].

Endoscopically, gastric lipomas typically appear as a soft, sharply defined, submucosal mass, often yellow in color as opposed to the surrounding pink mucosa. Typically, 3 clues help to identify these lesions as lipomas on endoscopic examination: the “tenting sign”, in which the overlying mucosa is easily retracted with the biopsy forceps; the “cushion sign” which occurs when the forceps produces a soft, cushioning indentation when applied to the lipoma; and the “naked fat” sign, which refers to exposed adipose tissue on the surface of the lipoma that pokes through the normal overlying mucosa after multiple biopsies of the normal mucosa are performed [6]. Occasionally, the he lesion may be associated with a centrally located, superficial ulceration, caused by pressure necrosis. At times, ulceration can be fairly extensive, leading to the false impression of a more aggressive lesion [4, 6]. Abdominal CT scan is the imaging examination of choice. It strongly suggests the diagnosis by showing a well-circumscribed lesion with a uniform, fatty density and an attenuation ranging from −70 to −120 H [4]. Endoscopic ultrasound examinations (EUS) is also a very good method for the diagnosis of gastric lipomas [2]. The typical findings of EUS reveal the tumor as an hyperechoic neoplasm in the submucosal layer.

Diagnosis is confirmed only after histopathological examination of surgical specimen. Standard biopsies are often inadequate because of the submucosal location of the tumor. On gross examination, gastric lipomas appear as solitary, smooth, soft masses, most often more than 2 cm in diameter, with an average size of 6.5 cm. On cut surface, they appear bright yellow, round, greasy, and encapsulated unless they have become infracted. Large lesions may be ulcerated, like in our patient [1, 2, 4]. Histologically, gastric lipomas are sharply circumscribed tumors, usually surrounded by a thick fibrous capsule. They are composed of mature adipocytes, relatively uniform in size and lacking cytologic atypia. The tumor is usually centered in the submucosa and often compresses the overlying muscularis mucosae [1, 7].

When the tumor is large, there is a progressive tendency for the submucosal mass to extrude into the lumen, leading to traumatic and inflammatory changes and resulting in necrosis, ulcération, and hemorrhage. Secondary changes including nuclear hypertrophy, hyperchromasia, fat necrosis, fatty cysts, and foamy macrophages may be observed. Lipoblasts and variation in cells size could, in addition, be present. The tumor can, in this situation, mimic a WDLS [3, 7, 8]. In such situation, diagnosis is difficult and morphological features are insufficient to classify the tumor. Cytogenetics and molecular biology offer new powerful tools for differentiating benign and malignant lipomatous tumors. WDLS are characterized by giant marker and ring chromosomes, sometimes as a sole finding or occasionally in association with other numerical or structural alterations. The giant marker and ring chromosomes contain amplified sequences of 12q13-15, the site of several genes (e.g., MDM2, GLI, SAS, CDK4, and HMGIC). This structural abnormality results in the consistent amplification of MDM2 and the frequent amplification of the adjacent genes, SAS, CDK4, and HMG1C [8]. The high specificity and sensitivity of detection of MDM2 and CDK4 amplification in WDLS and dedifferentiated liposarcomas have been demonstrated and analysis of these abnormalities using FISH or polymerase chain reaction has recently been shown to be an interesting means of identifying and separating WDSL from various benign lipomatous lesions [8–10].

The treatment of choice for a symptomatic gastric lipoma is circumferential excision with a clear margin of normal tissue. Due to the benign nature of this lesion an extensive surgical procedure such as an extended gastrectomy is not necessary. Simple local enucleation or partial gastric resection is sufficient to remove the lipoma without fear of relapses or malignant degeneration [2]. The prognosis of patients with gastric lipomas is good. Malignant degeneration has not been reported [2].

## Conclusion

Fatty tumors are rare in the gastrointestinal tract. Differentiating benign from malignant neoplasms is sometimes difficult in morphologic features. Cytogenetic procedures are, in these cases, the only means for an accurate diagnosis.
